# OMACC: an Optical-Map-Assisted Contig Connector for improving *de novo *genome assembly

**DOI:** 10.1186/1752-0509-7-S6-S7

**Published:** 2013-12-13

**Authors:** Yi-Min Chen, Chun-Hui Yu, Chi-Chuan Hwang, Tsunglin Liu

**Affiliations:** 1 Institute of Biotechnology, National Cheng Kung University, Tainan 70101, Taiwan; 2 Department of Engineering Science, National Cheng Kung University, Tainan 70101, Taiwan; 3 Supercomputing Research Center, National Cheng Kung University, Tainan 70101, Taiwan; 4 Institute of Bioinformatics and Biosignal Transduction, National Cheng Kung University, Tainan 70101, Taiwan

**Keywords:** *de novo *genome assembly, gap closer, optical map, contig graph

## Abstract

**Background:**

Genome sequencing and assembly are essential for revealing the secrets of life hidden in genomes. Because of repeats in most genomes, current programs collate sequencing data into a set of assembled sequences, called contigs, instead of a complete genome. Toward completing a genome, optical mapping is powerful in rendering the relative order of contigs on the genome, which is called scaffolding. However, connecting the neighboring contigs with nucleotide sequences requires further efforts. Nagarajian et al. have recently proposed a software module, FINISH, to close the gaps between contigs with other contig sequences after scaffolding contigs using an optical map. The results, however, are not yet satisfying.

**Results:**

To increase the accuracy of contig connections, we develop OMACC, which carefully takes into account length information in optical maps. Specifically, it rescales optical map and applies length constraint for selecting the correct contig sequences for gap closure. In addition, it uses an advanced graph search algorithm to facilitate estimating the number of repeat copies within gaps between contigs. On both simulated and real datasets, OMACC achieves a <10% false gap-closing rate, three times lower than the ~27% false rate by FINISH, while maintaining a similar sensitivity.

**Conclusion:**

As optical mapping is becoming popular and repeats are the bottleneck of assembly, OMACC should benefit various downstream biological studies via accurately connecting contigs into a more complete genome.

**Availability:**

http://140.116.235.124/~tliu/omacc

## Background

Genome assembly is essential for various downstream biological studies. *De novo *genome assembly, however, is still challenging mainly because of the presence of repeats in genomes [1]. It is even more daunting with next-generation-sequencing (NGS) data because NGS sequences, often called NGS reads, are shorter than traditional Sanger reads [2]. With shorter reads, more DNA segments become repeats. As a result, state-of-the-art assemblers turn NGS reads into a set of assembled sequences, called contigs, instead of one complete genome even for small microbes [3]. Although contigs already provide useful information, a complete genome is still superior because of its accurate and comprehensive genetic information [4].

**Figure 1:**
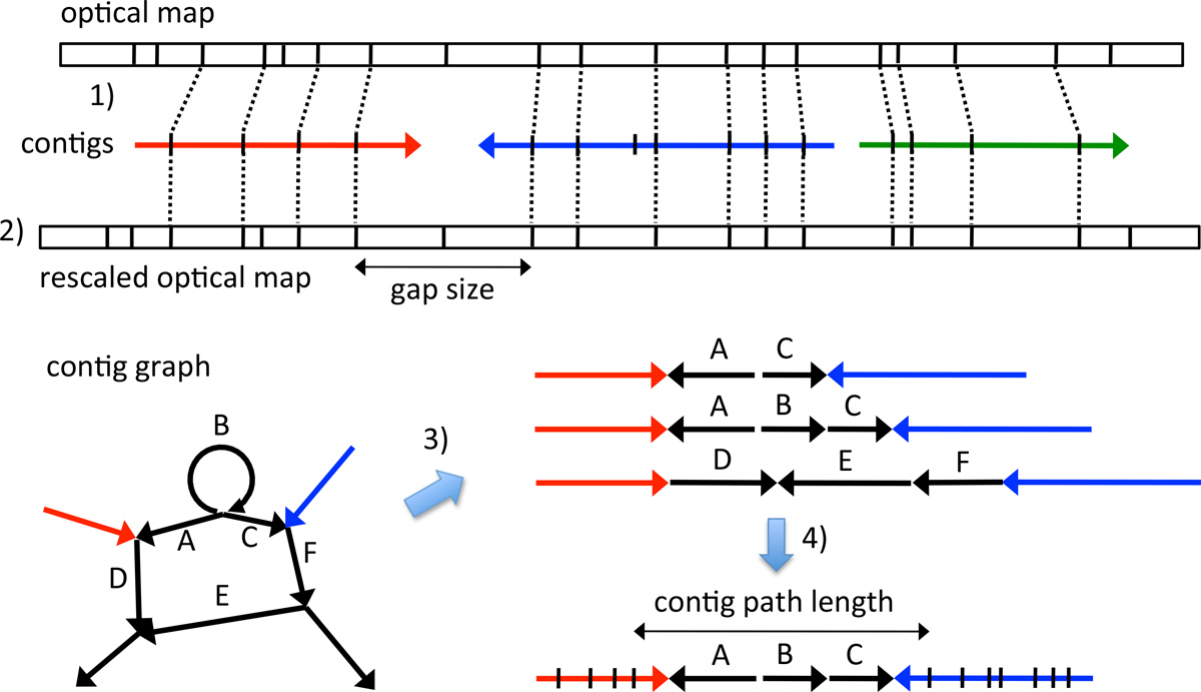
**OMACC workflow**. OMACC runs in four steps: 1) aligning contigs (colored lines with an arrow indicating the orientation) to the optical map (top rectangle) using SOMA2, and obtaining the relative order of contigs on the map, 2) rescaling optical map via comparing the lengths of restriction fragments on the map to the corresponding lengths on the contigs, 3) searching contig graph for all possible paths of contigs that connects each pair of neighboring contigs, and 4) determining the best path whose length is a closest match to the gap size.

Sequencing technologies keep evolving toward completing genomes. These include paired-end and mate-pair technologies [5], third generation sequencing [6], and optical mapping [7]. All the advances generate "long distance" information or longer reads to tackle repeat problems in genome assembly. Among these technologies, optical mapping is unique because its long distance information can go up to hundreds of kilobases (Kb) while other methods stay at the range of a few Kb. Unlike sequencing, optical mapping does not give bases. Instead, it digests a long DNA sequence into fragments of different lengths by a restriction enzyme, and collects the length information [8]. The resulting optical map allows one to align contigs to the map and infer their order on the genome (see Figure 1 for illustration). Completing genomes is then easier with the ordered contigs, also called scaffolds, as a backbone. Optical mapping was first proposed about 20 years ago [9], but the commercial machine by OpGen appeared only recently.

Scaffolding contigs, however, is not equivalent to completing a genome. One still needs to close the gaps between contigs with nucleotide sequences. Several gap closers have been proposed [10,11]. But they are not suitable for filling gaps in the scaffolds obtained via optical map because the gaps are often dozens of Kb in size and can be more than a hundred Kb long. For such big gaps, Nagarajan et al. proposed using other contig sequences for gap closures (their program called FINISH here) [12]. Given two neighboring contigs, FINISH searches the so-called contig graph (see Figure 1 for illustration) for a path of contigs that connects the two neighboring contigs. A contig graph describes all possible connections between contigs. As an example, from the contig graph in Figure 1 one learns that the 3' end of the red contig is connected to the 3' end of contig A and 5' end of contig D. Such connections suggest that the red contig is a repeat that appears twice in the genome and connects to two different sequences at its 3' end. When many repeats are present, contigs are usually interconnected in a complex way, forming a network of contigs, i.e., contig graph. This network reveals how one contig can be linked to another via other contigs. It is common that two or more paths of contigs exist between any two contigs. FINISH, however, does not pick the correct contig path for gap closure in some cases.

In this work, we present a computational program OMACC, an Optical-Map-Assisted Contig Connector. OMACC is advantageous because it takes into account gap size carefully via rescaling optical map and applying length constraint on selecting the path of contigs for gap closure. In addition, it applies an advanced graph search algorithm to efficiently infer the correct number of repeat copies in the gap between two contigs. We apply OMACC and FINISH on both simulated and real data sets. OMACC achieves a >90% accuracy, higher than the <73% by FINISH, and more than doubles the contig N50 lengths. OMACC also maintains a similar sensitivity as FINISH does. Thus, OMACC should benefit various downstream biological studies via accurately connecting contigs into a more complete genome with the assistance of optical map.

## Methods

### Data and assembly

The *E. coli *data came from the SOMA2 [13] package, which contained contig graph information ("454Contigs.ace") and two synthetic optical maps by the restriction enzymes AflII and NheI. We parsed the "454Contigs.ace" file to obtain all contig sequences, including those shorter than 500 bp. For *Myxosarcina sp*., GI1, we ran 454 sequencing and optical mapping on the genomic DNAs. The 454 sequencing was performed on GS FLX Titanium at Mission Biotech, Taiwan. The optical mapping was performed using the restriction enzyme AflII on the OpGen ARGUS system at Yourgene Biosciences, Taiwan. The optical mapping data were assembled by MapSolver (v0.5). All experiments were done following the manufacturers' protocols. We used Newbler [14] (v2.6) to assemble the 454 reads of GI1 into contigs and obtained the contig graph.

### OMACC workflow

OMACC requires contig sequences, the contig graph, and the optical map as input. With these data, it runs in four steps (Figure 1). First, OMACC aligns contigs to the optical map using SOMA2 [13] and obtains the relative order of contigs on the map. Second, it rescales the optical map via comparing the lengths of restriction fragments (RFs) on the map to the corresponding lengths on the contigs. In the third step, for each pair of neighboring contigs, OMACC searches the contig graph for all possible paths of contigs connecting the two neighboring contigs. Lastly, it determines the best contig path whose length is a closest match to the gap size. We describe each step in details below.

#### Aligning contigs to optical map

For each contig, SOMA2 computationally recognizes all the restriction sites and cleaves the contig into an ordered set of RFs. It then matches the RF sizes on the contig to those on the map (Figure 1). SOMA2 outputs both unique and non-unique matches. By default, OMACC closes the gaps between the uniquely aligned contigs because their relative positions on the map are of a higher confidence. OMACC also offers an option to include non-uniquely aligned contigs.

#### Rescaling optical map

**Figure 2:**
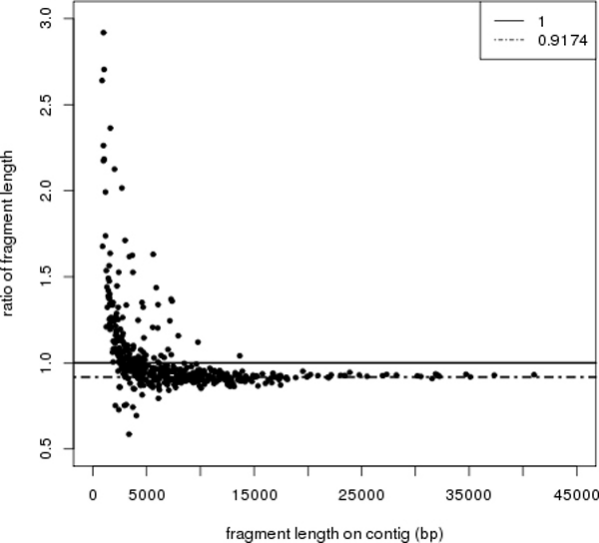
**Ratio of restriction fragement lengths on the optical map to those on the contig sequences (GI1 data)**. The ratio varies when the contig length is below 10 Kb. For contigs of length at least 10 Kb, a consistent ratio 0.9174 is obtained.

During alignment, sometimes two or more RFs on a contig are matched to one RF on the optical map (or vice versa), forming a so-called RF block (between two dashed lines in Figure 1). These are often the results of non-perfect enzyme restrictions or sequencing and assembly errors. OMACC selects the RF blocks with only one fragment on both the contig and the map. The length ratios were calculated (see Figure 2 for example) for deriving a rescaling factor. Because length ratio often varies a lot for small RFs, OMACC uses only the RFs at least 10 Kb long for deriving the rescaling factor, which is defined as the mean of the length ratios.

#### Searching contig graph

**Figure 3:**
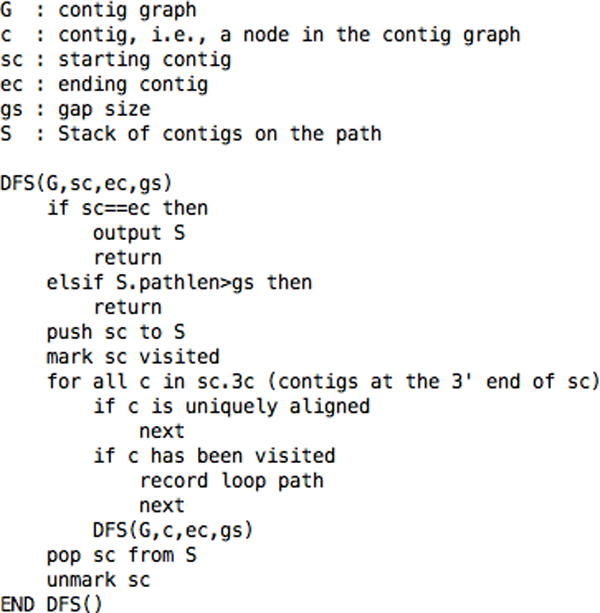
**Pseudo-code of our modified DFS algorithm**.

For each pair of neighboring contigs on the optical map, OMACC uses a modified depth-first-search (DFS) algorithm (Figure 3) to search the contig graph for all possible paths of contigs between the two neighboring contigs. The modified DFS algorithm considers contig orientations, i.e., contig connections at 5' or 3' end. More importantly, it applies length constraints, uniqueness of contig alignment, and a loop-path recording method to avoid deep recursion. OMACC defines the gap between two neighboring contigs as from the rightmost restriction site of the left contig to the leftmost site of the right contig (Figure 1). A gap size is the total length of the RFs within the gap. Note that when a RF on the map is shorter than 2 Kb, OMACC sets the RF length as 2 Kb. In our experiences, RFs shorter than 2 Kb may disappear from the map. Our modified DFS stops a search when the contig path length exceeds 1.2 fold of the gap size. It also stops a search when a uniquely aligned contig is encountered along the path because it should appear only once on the genome. Finally, it records a loop when encountering a contig that has been visited on the path and avoids searching the loop again. This avoids deep recursion and makes the algorithm more practical. The recorded loop paths will be used later for determining the number of repeat copies.

#### Determining best contig path

OMACC selects from all possible contig paths the one whose length is closest to the gap size. When a loop of contigs is detected along a contig path, the best number of loop copies resulting in a length closest to the gap size is calculated. When two or more loops of contigs are detected along a contig path, OMACC obtains the best number of loop copies for each loop. However, it does not consider the combination of loops to avoid the exponentially increasing number of combinations. In addition, OMACC requires the length difference between the two best contig paths to be greater than a cutoff (2Kb by default) to ensure the validity of the best contig path. Finally, when the best contig path length is within 2 Kb from the gap size, OMACC connects the two neighboring contigs using the sequences of the contigs on the best path.

## Results

### NGS data, optical map, and assembly

In this work, we analyzed the data of two species, *Escherichia coli *K12 and *Myxosarcina sp*., GI1, a cyanobacterium strain (Methods). The *E. coli *assembly contained 126 contigs, among which 88 were large (length ≥ 500 bp). The total length and N50 length of the large contigs were 4,552,797 bp and 112,233 bp, respectively. The contig graph contained 225 connections between 123 contigs and all the contigs were in the same connected component. The optical map of *E. coli *comprised 508 RFs with a total length 4,622 Kb.

The 454 sequencing of GI1 resulted in 440,104 reads with an average length 386 bp. We assembled the 454 reads into 267 contigs (Methods), among which 217 were large. The total length and N50 length of the large contigs were 6,997,371 bp and 84,919 bp, respectively. The contig graph contained 291 connections between 234 contigs. The majority, 220, of the 234 contigs were in the same connected component. The optical map assembly resulted in six maps with a total map size 7,289 Kb, similar to the total contig length. The largest map was 5,291 Kb in length and comprised 654 fragments, and was circular. We studied only the largest map in this work.

### Order of *E. coli *contigs

Before applying OMACC and FINISH to the *E. coli *data, we aligned the *E. coli *contigs to its known reference using BLAT [15] (option: -minMatch = 1) and determined the contig order as follows. On the reference genome, we first picked the longest contig alignment and excluded other alignments overlapping the longest one. This procedure was repeated for the next longest alignment until no alignment remained. Because some short contigs were missed during alignment, we checked all pairs of neighboring contigs with a ≥10 bp gap in between. We filled in these gaps if a short contig could be found matching the sequence at the gap. In addition, we checked the contigs that were not fully aligned and replaced it with another contig if that could be fully aligned to the position. The resulting alignments and order of contigs were shown in Table S1, Additional file 1. Among the 126 contigs, 118 were aligned to the reference genome. Most contigs were well aligned as only three had more than five unaligned base. The genome was covered almost completely (>99.96% aligned). Among the 118 contigs, 42 appeared twice or more times, indicating non-simple repeat structures. We used this contig order to evaluate the performance of *E. coli *contig connections.

### Connection of *E. coli *contigs

Among the 88 large contigs of *E. coli*, SOMA2 aligned 41 uniquely to the optical map (Table S2, Additional file 2). Since the *E. coli *genome is circular, there were 41 gaps between pairs of neighboring contigs. OMACC closed 22 (53.7%) gaps (Table 1) while FINISH made 30 (73.2%) gap closures (Table 2). Although FINISH closed eight more gaps, OMACC achieved a higher accuracy. That is, 20 of the 22 (91%) gaps closed by OMACC were correct. In contrast, FINISH only correctly closed 21 of the 30 (71%) gaps.

**Table 1:** Connections of the *E. coli *contigs by OMACC. For contigs, the number, "+/-", and ":" stand for the contig index, strand, and connections, respectively.

Neighboring contigs	Gap size (bp)	Best contig path	Contig path length (bp)	Length difference	Correct
006+,091+	2326	006+:116+:091+	2327	1	1

004+,086+	10658	004+:005+:086+	10660	2	1

032-,046+	6058	032-:047+:046+	6056	2	1

044-,017+	11342	044-:047+:017+	11340	2	1

013+,061+	23861	013+:011-:053+:061+	23864	3	1

010+,069-	4003	010+:078+:069-	4007	4	1

050-,059-	17496	050-:116-:059-	17500	4	1

012-,036-	20629	012-:011+:036-	20635	6	1

086+,054-	6739	086+:117-:054-	6745	6	1

017+,012-	10239	017+:018+:012-	10246	7	1

029-,010+	38377	029-:015+:056+:116-:010+	38384	7	1

057-,079-	29696	057-:053-:011+:075+:111-:080-:079-	29703	7	1

023+,022+	40884	023+:117+:022+	40893	9	1

022+,002+	49821	022+:116-:068-:011-:053+:081-:116-:002+	49831	10	1

048+,044-	27181	048+:028+:044-	27191	10	1

002+,050-	25463	002+:015+:050-	25475	12	1

108+,062-	48785	108+:117-:062-	48800	15	1

062-,016+	11600	062-:015-:016+	11238	362	1

091+,067-	24244	091+:053-:011+:067-	23876	368	1

076+,029-	10053	076+:028+:029-	9285	768	1

001+,023+	29005	001+:117+:023+	29788	783	0*

016+,033-	8649	016+:083+:033-	6842	1807	0*

The last column indicates whether the best contig path matches the true contig order in Table S1, Additional file 1.

*True contig path not in the contig graph.

**Table 2:** Connections of the *E. coli *contigs by FINISH. The notations are the same as in Table 1.

Neighboring contigs	Gap size (bp)	Best contig path	Contig path length (bp)	Length difference	Correct
006+,091+	2326	006+:116+:091+	2327	1	1

004+,086+	10658	004+:005+:086+	10660	2	1

032-,046+	6058	032-:047+:046+	6056	2	1

044-,017+	11342	044-:047+:017+	11340	2	1

013+,061+	23861	013+:011-:053+:061+	23864	3	1

010+,069-	4003	010+:078+:069-	4007	4	1

050-,059-	17496	050-:116-:059-	17500	4	1

012-,036-	20629	012-:011+:036-	20635	6	1

086+,054-	6739	086+:117-:054-	6745	6	1

017+,012-	10239	017+:018+:012-	10246	7	1

029-,010+	38377	029-:015+:056+:116-:010+	38384	7	1

057-,079-	29696	057-:053-:011+:075+:111-:080-:079-	29703	7	1

023+,022+	40884	023+:117+:022+	40893	9	1

048+,044-	27181	048+:028+:044-	27191	10	1

002+,050-	25463	002+:015+:050-	25475	12	1

108+,062-	48785	108+:117-:062-	48800	15	1

120+,105-	57776	120+:115-:043-:103-:039+:104+:113-:100-:073-:105-	57885	109	0

059-,107-	15847	059-:078+:063+:092+:110-:027-:107-	15609	238	0*

062-,016+	11600	062-:015-:016+	11238	362	1

091+,067-	24244	091+:053-:011+:067-	23876	368	1

046+,120+	18124	046+:101+:084-:087-:025-:122-:120+	18753	629	1

054-,032-	41348	054-:101+:084-:087-:025-:122-:123-:118-:116-:032-	41983	635	1

076+,029-	10053	076+:028+:029-	9285	768	1

001+,023+	29005	001+:117+:023+	29788	783	0*

016+,033-	8649	016+:083+:033-	6842	1807	0*

069-,108+	113278	069-:116-:074+:082+:084-:087-:025-:122-:123-:088+:026-:092+:110-:027-:108+	111423	1855	0*

045+,057-	56400	045+:115-:043-:121-:024-:005+:003-:058+:119+:057-	46042	10358	0*

022+,002+	49821	022+:116-:002+	14242	35579	0

038-,076+	144606	038-:117+:111+:075-:011-:053+:021+:124+:014-:015-:076+	86949	57657	0*

085+,048+	122463	085+:116+:007-:111-:048+	52387	70076	0*

For the two mis-connections by OMACC, "16+:83+:33-" and "1+:117+:23+", the correct contig paths were "16+:83-:34+:83+:33-" and "1+:23+", respectively (the number, "+/-", and ":" represented the contig index, strand, and connections, respectively). In these two cases, OMACC made mistakes simply because the correct path did not exist in the contig graph. That is, the connections "16+:83-" and "1+:23+" were missing in the contig graph. In fact, the differences between gap size and contig path length of the two mis-connections were the two largest ones (Table 1), suggesting their lower confidence.

Among the nine mis-connections by FINISH, the true contig paths of seven cases did not exist in the contig graph (Table 2). Although these errors could be partly attributed to the non-perfect contig graph, OMACC avoided five of the seven mis-connections using length constraints. In one of the two remaining cases, FINISH connected contigs "120+" and "105-" with "115-:43-:103-:39+:104+:113-:100-:73-" while the true contig path was "115-:43-:103-:39+:40+:114-:113-:100-:73-". In fact, OMACC found both paths, but did not select one for output because the two paths differed by only five bp in length. This indicates a possibility of incorrect gap closure by FINISH when two plausible paths exit. For the last mis-connection, "22+:116-:2+", the true contig path was "22+:116-:68-:11-:53+:81-:116-:2+". This path contained a loop of contigs and was correctly obtained by OMACC. Via this example, we demonstrate the robustness of OMACC in managing repeats for contig connections.

FINISH made two correct contig connections, "54-:101+:84-:87-:25-:122-:123-:118-:116-:32-" and "46+:101+:84-:87-:25-:122-:120+", that were not output by OMACC. In fact, the two contig paths were found by OMACC. However, OMACC also detected two other paths "54-:101+:84-:94-:25-:122-:123-:118-:116-:32-" and "46+:101+:84-:94-:25-:122-:120+", which were similar to the correct paths except that contig "87-" was replaced by "94-". Because the two contigs, "87-" and "94-", were equal in length, OMACC did not decide on the correct path. These pieces of information were recorded in the intermediate output of OMACC.

### Connections of GI1 contigs

Among the 217 large GI1 contigs, 61 were aligned uniquely to the optical map (Table S3, Additional file 3). OMACC and FINISH closed 21 (34.4%) and 22 (36.1%) of the gaps, respectively. Because no reference genome was currently available, we checked the difference between gap size and contig path length. By design, all the contig connections by OMACC were consistent, i.e., length difference less than 2 Kb (Table 3). In contrast, only 16 of the 22 (72.7%) gaps closed by FINISH were consistent (Table 4).

**Table 3:** Connections of GI1 contigs by OMACC.

Neighboring contigs	Gap size (bp)	Best contig path	Contig path length (bp)	Length difference	Consistent
010+,002+	6451	010+:245+:253+:245+:253+:245+:253+:245+:002+**	6444	7	1

019-,034+	24373	019-:200-:034+	24362	11	1

017+,043-	9345	017+:176-:043-	9365	20	1

018-,033-	21609	018-:262+:181-:259-:033-	21547	62	1

034+,007+	12182	034+:175-:007+	12096	86	1

003+,038+	16008	003+:227-:205+:038+	16095	87	1

015+,021-*	11178	015+:021-	11025	153	1

036+,013-	17106	036+:149-:013-	17269	163	1

039-,057-	16268	039-:175-:057-	16105	163	1

084+,071+	40296	084+:175+:142-:175+:142-:175+:142-:175+:142-:175+:142-:175+:142-:175+:071+**	40523	227	1

026+,044+	17568	026+:199+:193-:199+:044+**	17305	263	1

029-,070-	8250	029-:179-:070-	7930	320	1

035+,006-	67333	035+:261-:173-:099-:182+:006-	67009	324	1

023+,010+	19779	023+:159+:010+	20127	348	1

027-,016-	75414	027-:196+:152+:196+:103-:126-:063-:016-**	74985	429	1

037+,025+	18649	037+:176+:025+	18218	431	1

043-,056+	10828	043-:240+:056+	10288	540	1

004-,035+	24146	004-:175-:035+	23260	886	1

044+,020-	44467	044+:168+:020-	43369	1098	1

022-,026+*	11242	022-:026+	9992	1250	1

041+,104+	27829	041+:175-:142+:175-:142+:175-:104+**	29356	1527	1

The last column indicates whether the contig path length is consistent with the gap size, i.e., difference in length ≤2 Kb. *Gap closure unique to OMACC. **Best contig path different from those by FINISH.

**Table 4:** Connections of GI1 contigs by FINISH. Note that although FINISH does not rescale optical map, the rescaled gap sizes are shown here.

Neighboring contigs	Gap size (bp)	Best contig path	Contig path length (bp)	Length difference	Consistent
019-,034+	24373	019-:200-:034+	24362	11	1

017+,043-	9345	017+:176-:043-	9365	20	1

018-,033-	21609	018-:262+:181-:259-:033-	21547	62	1

034+,007+	12182	034+:175-:007+	12096	86	1

003+,038+	16008	003+:227-:205+:038+	16095	87	1

036+,013-	17106	036+:149-:013-	17269	163	1

039-,057-	16268	039-:175-:057-	16105	163	1

029-,070-	8250	029-:179-:070-	7930	320	1

035+,006-	67333	035+:261-:173-:099-:182+:006-	67009	324	1

023+,010+	19779	023+:159+:010+	20127	348	1

037+,025+	18649	037+:176+:025+	18218	431	1

043-,056+	10828	043-:240+:056+	10288	540	1

004-,035+	24146	004-:175-:035+	23260	886	1

010+,002+	6451	010+:245+:002+**	5556	895	1

044+,020-	44467	044+:168+:020-	43369	1098	1

026+,044+	17568	026+:199+:044+**	15881	1687	1

027-,016-	75414	027-:196+:103-:126-:063-:016-**	72109	3305	0

005+,008-*	10972	005+:175+:008-	17552	6580	0

041+,104+	27829	041+:175-:104+**	20998	6831	0

084+,071+	40296	084+:175+:071+**	15449	24847	0

030-,132+*	2838	030-:227-:205+:064+:159+:053-:175-:065-:132+	109213	106375	0

046-,119+*	23104	046-:230-:009-:154+:139-:101+:186-:105+:126-:048+:175+:053+:159-:064-:205-:227+:119+	322943	299839	0

*Gap closure unique to FINISH. **Best contig path different from those by OMACC.

OMACC and FINISH closed gaps for the same 19 pairs of neighboring contigs. However, the two methods made five different contig connections because of loops of contigs (Table 3 and 4). For example, between contigs "26+" and "44+", the contig paths obtained by OMACC and FINISH were "26+:199+:193-:199+:44+" and "26+:199+:44+", respectively, and differed by a loop. The contig path length with the loop (17,305 bp) was closer to the rescaled gap size on the optical map (17,568 bp) than the contig path length without the loop (15,881 bp). The path with the loop was further supported by read coverage. The 454 read coverage of the contigs "26", "193", and "44" were 24.7, 26.2, and 25.6X, respectively (data not shown). The contig "199" had a coverage 44.9X, indicating two copies of "199" in the GI1 genome. Thus, we demonstrate with real data that OMACC is more robust in managing repeats.

### Including non-uniquely aligned contigs

On the *E. coli *data, OMACC closed two more gaps, "60+:116+:85+" and "118-:116-:32-" (Table 5a), when including the nine non-uniquely aligned contigs (Table S2, Additional file 2). The true contig path of the first gap, "60+:85+", did not exist in the contig graph. Consistently, its difference between gap size and contig path length was the second largest. The second contig connection was correct. When including non-uniquely aligned contigs, FINISH closed seven more gaps (Table 5b). However, five original contig connections disappeared because the non-uniquely aligned contigs disrupted the original neighboring contig pairs. For example, contigs "81+" and "52-" were inserted between "38+" and "76-" (Table S2, Additional file 2). This disruption should not be problematic if "81+" and "52-" were in the contig path from "38+" to "76-", which did not hold true. In this case and many similar ones, both contig connections before and after adding non-uniquely aligned contigs were incorrect. One disruption, contig "118-" inserted between "54-" and "32-", was acceptable because "118-" was on the contig path from "54-" to "32-". In this case, the contig connection remained correct. Overall, adding non-uniquely aligned contigs did not alter much the performance of OMACC and FINISH on the *E. coli *data.

**Table 5:** Differences in contig connections before and after (indicating by "<" and ">" in the first column, respectively) including non-uniquely aligned contigs for (a) OMACC on *E. coli *data, (b) FINISH on *E. coli *data, (c) OMACC on GI1 data, and (d) FINISH on GI1 data.

In	Neighboring contigs	Gap size (bp)	Best contig path	Contig path length (bp)	Length difference	Correct/Consistent
(a)						

>	118-,032-	9331	118-:116-:032-	9334	3	1

>	060+,085+	6070	060+:116+:085+	7271	1201	0

(b)						

>	118-,032-	9331	118-:116-:032-	9334	3	1

<	059-,107-	15847	059-:078+:063+:092+:110-:027-:107-*	15609	238	0

>	054-,118-	13029	054-:101+:084-:087-:025-:122-:123-:118-	13656	627	1

<	054-,032-	41348	054-:101+:084-:087-:025-:122-:123-:118-:116-:032-	41983	635	1

>	060+,085+	6070	060+:116+:085+	7271	1201	0

<	069-,108+	113278	069-:116-:074+:082+:084-:087-:025-:122-:123-:088+:026-:092+:110-:027-:108+	111423	1855	0

>	112+,066+	10905	112+:116-:065+:117+:111+:066+	14522	3617	0

>	066+,048+	45302	066+:117+:111+:048+	19187	26115	0

>	081+,052-	68596	081+:053-:011+:051-:052-	39775	28821	0

<	038-,076+	144606	038-:117+:111+:075-:011-:053+:021+:124+:014-:015-:076+	86949	57657	0

>	038-,081+	36179	038-:117+:089-:015+:014+:124-:021-:053-:011+:068+:116+:081+	103480	67301	0

<	085+,048+	122463	085+:116+:007-:111-:048+	52387	70076	0

(c)						

>	014+,069+	6852	014+:069+	6761	91	1

<	035+,006-	67333	035+:261-:173-:099-:182+:006-	67009	324	1

<	027-,016-	75414	027-:196+:152+:196+:103-:126-:063-:016-	74985	429	1

>	048+,060-	55177	048+:175+:142-:175+:142-:175+:142-:175+:142-:175+:142-:175+:060-	55737	560	1

(d)						

<	035+,006-	67333	035+:261-:173-:099-:182+:006-	67009	324	1

<	027-,016-	75414	027-:196+:103-:126-:063-:016-	72109	429	0

>	104+,074+	35095	104+:175+:112-:167+:102+:258+:160-:136+:198-:225+:228+:074+	37882	2787	0

>	048+,060-	55177	048+:175+:060-	34842	20335	0

>	111+,006-	25002	111+:188-:149-:115-:173-:099-:182+:006-	52888	27886	0

<	046-,119+	23104	046-:230-:009-:154+:139-:101+:186-:105+:126-:048+:175+:053+:159-:064-:205-:227+:119+*	322943	299839	0

*These contig connections are not disrupted by any non-uniquely aligned contig, but disappear when non-uniquely aligned contigs are included, indicating a bug in FINISH.

On GI1 data, OMACC closed two more gaps while disrupted two contig connections (Table 5c). Again, all the contig connections of these altered cases were consistent in length by design. FINISH closed three more gaps and disrupted three contig connections (Table 5d). All the new contig connections and two of the three disrupted ones were inconsistent in length. Thus, adding the non-uniquely aligned contigs reduced one consistent contig connection by FINISH.

## Discussion

### OMACC and FINISH

The major differences between OMACC and FINISH are that FINISH does not take into account length information in an optical map and it does not find all possible paths between contigs. We find that alternative contig paths and loops of contigs in gaps are not uncommon. Because FINISH outputs the first path of contigs detected, the correct one may not be selected when alternative contig paths exist. In contrast, OMACC filters the contig path whose path length is not consistent with the gap size, increasing the chance for the correct contig paths to be selected. Note that if alternative contig paths still exist after filtering, OMACC does not close the gap; instead it outputs the paths to an intermediate file. These pieces of information can be helpful if additional information is available. For example, between the two *E. coli *contigs "46+" and "120+", OMACC finds two paths "101+:84-:87-:25-:122-" and "101+:84-:94-:25-:122-", identical in length and differed by one contig. On the reference genome, "94-" is on a path "82+:84-:94-:25-:122-" (Table S1, Additional file 1). If paired-end or mate-pair information supports only the proximity between "101+" and "87-", then one can close this gap correctly.

In terms of algorithm, OMACC is also more comprehensive. FINISH avoids deep recursion by limiting the number of contigs (default 15) on the searched path. In contrast, OMACC records all loop structures and avoids visiting them again. This allows OMACC to deal with long paths of contigs, which appear often because of nested or tandem repeats. Combining with length constraints, OMACC in principle can return all possible combinations of repeat structures. But it does not do so because when two or more loops of contigs exist, alternative contig paths are often found, leading to unclosed gaps. In any case, as repeat problem is the bottleneck of complete genome assembly, OMACC should move assembly closer toward complete than FINISH.

### Completeness of contig graph

On the *E. coli *data, OMACC and FINISH made two and nine mis-connections, among which the true contig paths did not exist in the contig graph in two and seven cases, respectively. Detailed investigations reveal three major reasons for the absence of the true contig paths. First, some true contig paths are not continuous on the genome. For example, the true contig path between "85+" and "48+" is "85+:116+:112-:18-:52+:95-:51+:11-:53+:125+:80+:111+:48+" (Table S1, Additional file 1). Along this path, all the neighboring contigs connect without gaps (>2 bp) except "52+" and "95-", in-between which a gap of 166 bp exists. It is likely that this gap region is not sequenced at all. As a result, the two contigs "52+" and "95-" are not connected in the contig graph. Strictly speaking, the path should not be called a true contig path because there should be a contig between "52+" and "95-", but the contig is missing. This scenario applies for the neighboring contigs "74+:30+", "93-:92+", and "52+:95-". Second, some neighboring contigs connect well (e.g., "93-:110-") or even overlap (e.g., "38-:99-") on the genome (Table S1, Additional file 1), but their connections are not indicated in the contig graph. Checking the assembly ACE file, we found that no read can bridge the two neighboring contigs long enough for them to be connected by assembler. Again, this indicates the depletion of 454 reads at the junctions between the contigs. The missing connections of "38-:99-", "1+:23+", "93-:110-", and "73-:119+" in the contig graph fall into this category. The above two types of missing connections may be avoided if the sequencing depth is increased. In the third scenario, we found that the contig connections indeed exist in the assembly ACE file, but the SOMA2 script "get_graph.pl" failed to parse them out. When a contig is flanked by the same repeat, e.g., "83-:34+:83+", "get_graph.pl" only outputs one of the connections between the two contigs. This happens in two other cases, "92+:109+:92+" and "43+:121+:43-". Thus, "get_graph.pl" should be used with caution. On Newbler assembly, we thus recommend using the "454ContigGraph.txt" file instead of parsing the contig graph from ACE file when applying SOMA. Note that OMACC parses "454ContigGraph.txt" directly for contig graph.

We further tested whether restoring these missing connections improved performance. Indeed, adding the missing connections eliminated the two mis-connections made by OMACC (data not shown). That is, OMACC became error-free with a comprehensive contig graph. In contrast, FINISH made four more connections, among which one was incorrect (data not shown). The overall accuracy of FINISH still remained as 71%. In general, we expect an even higher accuracy by OMACC, but not by FINISH, if the contig graph is improved.

### Rescaling optical map

Rescaling optical map can alter contig connections by OMACC. On the GI1 data, OMACC determined the best contig path between "26+" and "44+" as "26+:199+:193-:199+:44+", which contained a loop of contigs. Without rescaling, the gap size dropped from 17,305 bp to 15,876 bp, compared with which "26+:199+:44+" would be selected as the best contig path. Supported by read coverage, there should be two copies of "199+" in the genome. This example shows the importance and validity of our map rescaling.

On the rescaled map, we observed a trend that optical map claims longer RFs when the RFs are shorter (below 5 Kb). In principle, we can apply length dependent rescaling factors. However, the not uncommon large deviations from the fitted curve lower the confidence on length inference. It will be ideal if a mechanic model can be proposed to explain the trend, and the deviations can be explained in the future. Note that most gaps between pairs of neighboring contigs are longer than 5 Kb (38 of the 41 gaps and 57 of the 61 gaps for *E. coli *and GI1, respectively). Thus, our rescaled gap sizes should be accurate in most cases.

### Read coverage

It is possible to apply read coverage as another constraint on the number of repeats in the genome. When more than one repeat contig appears on a path, this may resolve the concern of exponentially increasing number of combinations. However, read coverage can fluctuate across genomes. For example, GC content has been known to affect read coverage [16]. Although the effects of GC bias can be tuned, the fluctuations still seem too noisy to render accurate estimation of copy numbers.

### Including non-uniquely aligned contigs

Although non-uniquely aligned contigs did not alter much the performance of OMACC and FINISH on *E. coli *and GI1 data, we found them potentially harmful. In the *E. coli *case, seven of the nine non-uniquely aligned contigs (Table S2, Additional file 2) are not properly placed on the genome (Table S1, Additional file 1). In contrast, all the uniquely aligned contigs are correctly placed. Although this may not be a good example since the *E. coli *optical map is synthetic and the data probably has been optimized, it is certain that uniquely aligned contigs are more likely to be placed correctly on the genome. Thus, we decide to turn off this option by default. However, this option can be useful for OMACC. For example, combining the GI1 results before and after including non-uniquely aligned contigs gave two more consistent contig connections. Note that the newly closed gaps did not disrupt any original contig connections because they occurred at different loci on the genome (Table 5 S3, Additional file 3). That is, the newly closed gaps were consistent with all original contig connections.

### Performance on Illumina data

In addition to 454 data, we also studied the performance of OMACC and FINISH on Illumina data. For comparison, real Illumina paired-end reads of *E. coli *K12 MG1655 were obtained from NCBI SRA [17] (accession SRX131053). We used Velvet [18,19] to assemble the Illumina data and optimized the assembly for fewer contigs and longer N50 length via scanning kmer values. The optimized assembly contained 89 large contigs (≥500 bp) and the N50 length was 132,586 bp, which were comparable to the statistics of Newbler assembly of the *E. coli*'s 454 data. On this dataset, however, OMACC and FINISH could not close any gap between contigs. Detailed investigation revealed that only seven contigs were uniquely aligned to the optical map (data not shown), and all the seven contigs were considered to be on different scaffolds by SOMA2. Compared with the 454 contigs, we found many more small insertions or deletions in the Illumina contigs, which could explain the worse alignments at least partly. This raises the possibility that the SOMA2 alignments may be improved if the small assembly errors can be reduced using Illumina reads with fewer errors. For this concern, we simulated several Illuimina PE libraries without any errors and repeated the analysis. Still, OMACC and FINISH could not close any gap on the perfect Illumina data (data not shown). Thus, even though the 454 and Illumina assemblies are comparable, optical map is much more useful for improving the 454 assembly than the Illumina assembly of *E. coli*. Note that it is still possible that optical map can improve Illumina assembly if other assemblers, e.g., ALLPATHS-LG, are used. However, we did not try those because many Illumina assemblers do not output the contig graph information.

### Challenges on complex eukaryotic genomes

In principle, OMACC should also benefit the finishing of complex eukaryotic genomes. However, there still exist practical challenges. First, SOMA2 alignment takes a much longer time when treating a large genome. For example, the SOMA2 alignment of the *E. coli *and GI1 contigs finished in a few minutes. However, for an ~11Mb genome, the alignment took about 16 hours (data not shown). For a genome of ~24 Mb, the alignment cannot be finished within a week. Thus, efficient alignment of contigs to optical map is necessary for treating eukaryotic genomes. Fortunately, this is possible because SOMA2 applies dynamic programming for alignments, which can be speed up using seed matching. Second, the current version of SOMA2 treats only two maps at most. This seems problematic because complex eukaryotes often have more than two chromosomes. One solution is to separate chromosomes by pulse field gradient gel electrophoresis before doing optical maps, which has been shown effective [20].

### Applicability

OMACC is written in Perl and requires SOMA2. By default, OMACC takes as input contig sequences in FASTA format and a contig graph in Newbler format ("454ContigGraph.txt"). OMACC also offers a script to convert the optical map file generated by OpGen, currently the most popular optical mapping platform, into the required format. Although OMACC does not improve the Velvet assembly of the *E. coli*, it may works for other species. Thus, OMACC still contains scripts to treat contigs and contig graph generated by Velvet. Finally, OMACC holds a script to connect the contig sequences together. With this script, OMACC raised the contig N50 length of *E. coli *and GI1 from 112,233 bp to 294,887 bp and from 84,919 bp to 180,233 bp, respectively.

## Conclusion

Toward completing a genome, optical map is helpful in scaffolding contigs. However, many scientists still have to conduct experiments and manually fill in the gaps between the scaffolded contigs. To reduce these costly efforts, we present a computational program OMACC that closes the gaps between scaffolded contigs with a higher accuracy compared with a similar tool. As accuracy is of a great concern at the stage of completing genome, OMACC is useful for finishing genomes. We expect OMACC to benefit many scientists because genome assembly is the fundamental of many biological studies.

## Competing interests

The authors declare that they have no competing interests.

## Authors' contributions

YMC performed sequencing and optical mapping experiments, and wrote the paper. CHY conducted data analysis. CCH wrote the paper. TL conceived OMACC, conducted analysis, and wrote the paper.

## Supplementary Material

Additional file 1 Table S1. Alignments of the *E. coli *contigs on the reference genome (gi|49175990|ref|NC_000913.2|) sorted by alignment start on the reference genome. Click here for file

Additional file 2 Table S2. Order of *E. coli *contigs inferred from the SOMA2 alignments and the mapping status (U: unique, N: non-unique). Click here for file

Additional file 3 **Table S3. Order of GI1 contigs inferred from the SOMA2 alignments and the mapping status (U: unique, N: non-unique)**. Click here for file

## Declarations

The publication fee is supported by National Science Council, Taiwan (NSC 102-2627-B-006-003).

This article has been published as part of *BMC Systems Biology *Volume 7 Supplement 6, 2013: Selected articles from the 24th International Conference on Genome Informatics (GIW2013). The full contents of the supplement are available online at http://www.biomedcentral.com/bmcsystbiol/supplements/7/S6.
