# Supplementary material for: OMACC: an Optical-Map-Assisted Contig Connector for improving de novo genome assembly
Source: BMC Syst Biol. 2013 Dec 13;7(Suppl 6):S7. doi: 10.1186/1752-0509-7-S6-S7 (PMC4029551; doi:10.1186/1752-0509-7-S6-S7)
Supplement: Additional file 1 — Table S1. Alignments of the E. coli contigs on the reference genome (gi|49175990|ref|NC_000913.2|) sorted by alignment start on the reference genome. [file 1752-0509-7-S6-S7-S1.docx]

| Contig | Strand | Contig length (bp) | Contig alignment start | Contig alignment end | Reference alignment start | Reference alignment end | Mismatches | Gap bases |
| --- | --- | --- | --- | --- | --- | --- | --- | --- |
| 079 | - | 148241 | 0 | 15389 | 0 | 15387 | 0 | 2 |
| 078 | + | 1345 | 0 | 1345 | 15387 | 16732 | 0 | 0 |
| 064 | - | 3063 | 0 | 3063 | 16732 | 19795 | 0 | 0 |
| 117 | + | 768 | 0 | 768 | 19795 | 20563 | 0 | 0 |
| 031 | + | 105517 | 0 | 105516 | 20563 | 126079 | 0 | 2 |
| 020 | - | 38295 | 0 | 38295 | 126315 | 164609 | 0 | 3 |
| 055 | - | 59164 | 0 | 59164 | 164579 | 223743 | 0 | 0 |
| 103 | - | 210 | 0 | 210 | 223743 | 223953 | 0 | 0 |
| 039 | + | 818 | 0 | 818 | 223953 | 224771 | 0 | 0 |
| 104 | + | 864 | 0 | 864 | 224771 | 225635 | 1 | 0 |
| 113 | - | 85 | 0 | 85 | 225636 | 225721 | 1 | 0 |
| 100 | - | 16 | 0 | 16 | 225721 | 225738 | 1 | 1 |
| 073 | - | 3010 | 0 | 3010 | 225738 | 228749 | 6 | 1 |
| 119 | + | 121 | 0 | 121 | 228750 | 228871 | 1 | 0 |
| 038 | - | 29036 | 0 | 29036 | 228871 | 257907 | 0 | 0 |
| 099 | - | 11865 | 0 | 11865 | 257899 | 269764 | 0 | 0 |
| 080 | + | 1041 | 0 | 1041 | 269764 | 270804 | 1 | 1 |
| 111 | + | 181 | 0 | 181 | 270804 | 270985 | 1 | 0 |
| 007 | + | 2192 | 0 | 2192 | 270986 | 273178 | 0 | 0 |
| 116 | - | 1195 | 0 | 1195 | 273178 | 274373 | 0 | 0 |
| 065 | + | 4013 | 0 | 4013 | 274373 | 278386 | 0 | 0 |
| 117 | + | 768 | 0 | 768 | 278386 | 279154 | 9 | 0 |
| 111 | + | 181 | 0 | 181 | 279154 | 279335 | 0 | 0 |
| 066 | + | 10521 | 0 | 10521 | 279335 | 289856 | 0 | 0 |
| 117 | + | 768 | 0 | 768 | 289857 | 290625 | 9 | 0 |
| 089 | - | 23827 | 0 | 23827 | 290625 | 314452 | 0 | 0 |
| 015 | + | 1255 | 0 | 1255 | 314452 | 315707 | 0 | 0 |
| 014 | + | 33212 | 0 | 33212 | 315707 | 348918 | 0 | 1 |
| 124 | - | 92 | 0 | 92 | 349011 | 349103 | 1 | 0 |
| 124 | - | 92 | 0 | 92 | 349104 | 349196 | 0 | 0 |
| 021 | - | 31186 | 0 | 31186 | 349196 | 380483 | 0 | 101 |
| 053 | - | 625 | 0 | 625 | 380483 | 381108 | 0 | 0 |
| 011 | + | 706 | 0 | 706 | 381108 | 381814 | 0 | 0 |
| 049 | - | 9116 | 0 | 9116 | 381815 | 390931 | 0 | 0 |
| 015 | - | 1255 | 0 | 1255 | 390932 | 392187 | 0 | 0 |
| 076 | + | 132773 | 0 | 132773 | 392187 | 524960 | 0 | 6 |
| 028 | + | 983 | 0 | 983 | 524960 | 525943 | 0 | 0 |
| 029 | - | 40061 | 0 | 40061 | 525943 | 566002 | 1 | 2 |
| 015 | + | 1255 | 0 | 1255 | 566002 | 567257 | 0 | 0 |
| 056 | + | 6555 | 0 | 6555 | 567258 | 573813 | 0 | 0 |
| 116 | - | 1195 | 0 | 1195 | 573813 | 575008 | 0 | 0 |
| 010 | + | 32224 | 0 | 32224 | 575008 | 607230 | 0 | 2 |
| 078 | + | 1345 | 0 | 1344 | 607230 | 608574 | 0 | 0 |
| 069 | - | 78496 | 0 | 78496 | 608576 | 687073 | 0 | 1 |
| 116 | - | 1195 | 0 | 1195 | 687073 | 688268 | 0 | 0 |
| 074 | + | 7619 | 0 | 7619 | 688268 | 695887 | 0 | 0 |
| 030 | + | 283 | 0 | 283 | 695997 | 696280 | 0 | 0 |
| 082 | + | 32744 | 0 | 32744 | 696280 | 729025 | 0 | 1 |
| 084 | - | 733 | 0 | 733 | 729025 | 729758 | 2 | 0 |
| 094 | - | 1470 | 0 | 1470 | 729758 | 731228 | 0 | 0 |
| 025 | - | 598 | 0 | 598 | 731228 | 731826 | 6 | 0 |
| 122 | - | 86 | 0 | 86 | 731826 | 731912 | 0 | 0 |
| 123 | - | 65 | 0 | 65 | 731912 | 731977 | 0 | 0 |
| 088 | + | 207 | 0 | 207 | 731977 | 732184 | 1 | 0 |
| 102 | - | 1513 | 0 | 1513 | 732184 | 733697 | 0 | 0 |
| 122 | - | 86 | 0 | 86 | 733697 | 733783 | 0 | 0 |
| 123 | - | 65 | 0 | 65 | 733783 | 733848 | 0 | 0 |
| 088 | + | 207 | 0 | 207 | 733848 | 734055 | 0 | 0 |
| 026 | - | 45932 | 0 | 45932 | 734055 | 779987 | 0 | 0 |
| 092 | + | 80 | 1 | 78 | 779987 | 780064 | 0 | 0 |
| 110 | - | 76 | 0 | 76 | 780064 | 780140 | 0 | 0 |
| 093 | - | 147 | 48 | 147 | 780140 | 780239 | 0 | 0 |
| 092 | + | 80 | 1 | 79 | 780290 | 780368 | 0 | 0 |
| 110 | - | 76 | 0 | 76 | 780368 | 780444 | 0 | 0 |
| 093 | - | 147 | 0 | 147 | 780444 | 780591 | 5 | 0 |
| 110 | - | 76 | 0 | 75 | 780591 | 780666 | 0 | 0 |
| 027 | - | 194 | 0 | 194 | 780874 | 781067 | 0 | 3 |
| 108 | + | 267929 | 0 | 267929 | 781068 | 1048999 | 0 | 4 |
| 117 | - | 768 | 0 | 768 | 1049000 | 1049768 | 9 | 0 |
| 062 | - | 43700 | 0 | 43700 | 1049768 | 1093467 | 0 | 1 |
| 015 | - | 1255 | 0 | 1255 | 1093467 | 1094722 | 0 | 0 |
| 016 | + | 111931 | 0 | 111931 | 1094722 | 1207012 | 0 | 365 |
| 083 | - | 16 | 0 | 16 | 1207012 | 1207028 | 0 | 0 |
| 034 | + | 1797 | 0 | 1797 | 1207028 | 1208825 | 0 | 0 |
| 083 | + | 16 | 0 | 16 | 1208825 | 1208841 | 0 | 0 |
| 033 | - | 77170 | 0 | 77170 | 1208841 | 1286010 | 0 | 1 |
| 097 | - | 111 | 0 | 111 | 1286010 | 1286121 | 0 | 0 |
| 097 | - | 111 | 0 | 102 | 1286197 | 1286299 | 6 | 0 |
| 060 | + | 12176 | 0 | 12176 | 1286545 | 1298721 | 0 | 0 |
| 085 | + | 95349 | 0 | 95349 | 1298717 | 1394067 | 0 | 1 |
| 116 | + | 1195 | 0 | 1195 | 1394067 | 1395262 | 0 | 0 |
| 112 | - | 35420 | 0 | 35420 | 1395262 | 1430682 | 0 | 2 |
| 018 | - | 1688 | 0 | 1688 | 1430682 | 1432370 | 3 | 0 |
| 052 | + | 31999 | 0 | 31999 | 1432370 | 1464369 | 0 | 0 |
| 095 | - | 128 | 0 | 128 | 1464535 | 1464663 | 0 | 0 |
| 051 | + | 1270 | 0 | 1270 | 1464663 | 1465933 | 0 | 0 |
| 011 | - | 706 | 0 | 706 | 1465933 | 1466639 | 0 | 0 |
| 053 | + | 625 | 0 | 625 | 1466639 | 1467264 | 0 | 0 |
| 125 | + | 53 | 0 | 53 | 1467266 | 1467319 | 0 | 0 |
| 080 | + | 1041 | 0 | 1041 | 1467319 | 1468359 | 0 | 1 |
| 111 | + | 181 | 0 | 181 | 1468359 | 1468540 | 0 | 0 |
| 048 | + | 57673 | 0 | 57673 | 1468540 | 1526214 | 0 | 1 |
| 028 | + | 983 | 0 | 983 | 1526214 | 1527197 | 4 | 0 |
| 044 | - | 41495 | 0 | 41495 | 1527197 | 1568692 | 0 | 0 |
| 047 | + | 1133 | 0 | 1133 | 1568693 | 1569826 | 3 | 0 |
| 017 | + | 61147 | 0 | 61147 | 1569826 | 1630973 | 0 | 2 |
| 018 | + | 1688 | 0 | 1688 | 1630973 | 1632661 | 2 | 0 |
| 012 | - | 16205 | 0 | 16205 | 1632661 | 1648866 | 0 | 0 |
| 011 | + | 706 | 0 | 706 | 1648866 | 1649572 | 0 | 0 |
| 036 | - | 57066 | 0 | 57066 | 1649572 | 1706637 | 0 | 1 |
| 037 | + | 96 | 0 | 96 | 1706637 | 1706733 | 0 | 0 |
| 037 | + | 96 | 0 | 96 | 1706733 | 1706829 | 1 | 0 |
| 001 | + | 164204 | 0 | 164204 | 1706860 | 1871063 | 0 | 1 |
| 023 | + | 105470 | 0 | 105470 | 1871055 | 1976525 | 0 | 0 |
| 117 | + | 768 | 0 | 768 | 1976526 | 1977294 | 0 | 0 |
| 022 | + | 86887 | 0 | 86887 | 1977295 | 2064181 | 0 | 1 |
| 116 | - | 1195 | 0 | 1195 | 2064182 | 2065377 | 5 | 0 |
| 068 | - | 1586 | 0 | 1586 | 2065378 | 2066964 | 0 | 0 |
| 011 | - | 706 | 0 | 706 | 2066964 | 2067670 | 0 | 0 |
| 053 | + | 625 | 0 | 625 | 2067670 | 2068295 | 0 | 0 |
| 081 | - | 31477 | 0 | 31477 | 2068295 | 2099772 | 0 | 0 |
| 116 | - | 1195 | 0 | 1195 | 2099772 | 2100967 | 0 | 0 |
| 002 | + | 67231 | 0 | 67231 | 2100967 | 2168197 | 0 | 1 |
| 015 | + | 1255 | 0 | 1255 | 2168197 | 2169452 | 0 | 0 |
| 050 | - | 117493 | 0 | 117493 | 2169452 | 2286940 | 0 | 5 |
| 116 | - | 1195 | 0 | 1195 | 2286940 | 2288135 | 0 | 0 |
| 059 | - | 223711 | 0 | 223711 | 2288135 | 2512295 | 0 | 455 |
| 078 | + | 1345 | 0 | 1345 | 2512295 | 2513640 | 0 | 0 |
| 063 | + | 5310 | 0 | 5310 | 2513640 | 2518950 | 0 | 0 |
| 092 | + | 80 | 0 | 79 | 2518950 | 2519031 | 0 | 2 |
| 109 | + | 41 | 0 | 41 | 2519031 | 2519072 | 0 | 0 |
| 092 | + | 80 | 1 | 80 | 2519072 | 2519151 | 0 | 0 |
| 110 | - | 76 | 0 | 76 | 2519273 | 2519349 | 0 | 0 |
| 027 | - | 194 | 0 | 194 | 2519349 | 2519542 | 0 | 1 |
| 107 | - | 204550 | 0 | 204550 | 2519542 | 2724094 | 0 | 6 |
| 119 | - | 121 | 0 | 121 | 2724094 | 2724215 | 1 | 0 |
| 073 | + | 3010 | 0 | 3010 | 2724216 | 2727226 | 8 | 10 |
| 113 | + | 85 | 0 | 85 | 2727234 | 2727319 | 1 | 0 |
| 042 | + | 274 | 0 | 274 | 2727319 | 2727593 | 0 | 0 |
| 096 | - | 24 | 0 | 24 | 2727593 | 2727617 | 0 | 0 |
| 040 | - | 561 | 0 | 561 | 2727617 | 2728178 | 1 | 0 |
| 039 | - | 818 | 0 | 818 | 2728178 | 2728996 | 3 | 0 |
| 013 | + | 264751 | 0 | 264751 | 2728996 | 2994381 | 0 | 642 |
| 011 | - | 706 | 0 | 706 | 2994382 | 2995088 | 0 | 0 |
| 053 | + | 625 | 0 | 625 | 2995088 | 2995713 | 0 | 0 |
| 061 | + | 48359 | 0 | 48359 | 2995715 | 3044073 | 0 | 3 |
| 006 | + | 84127 | 5 | 84127 | 3044046 | 3128167 | 0 | 1 |
| 116 | + | 1195 | 0 | 1195 | 3128167 | 3129362 | 0 | 0 |
| 091 | + | 54754 | 0 | 54754 | 3129362 | 3184116 | 0 | 0 |
| 053 | - | 625 | 0 | 625 | 3184117 | 3184742 | 0 | 0 |
| 011 | + | 706 | 0 | 706 | 3184742 | 3185448 | 0 | 0 |
| 067 | - | 51857 | 0 | 51857 | 3185448 | 3237682 | 0 | 377 |
| 019 | - | 125799 | 0 | 125799 | 3237663 | 3363577 | 2 | 117 |
| 116 | - | 1195 | 0 | 1195 | 3363577 | 3364772 | 0 | 0 |
| 008 | - | 25398 | 0 | 25398 | 3364772 | 3390169 | 0 | 1 |
| 009 | + | 91 | 0 | 91 | 3390169 | 3390260 | 0 | 0 |
| 009 | + | 91 | 0 | 91 | 3390260 | 3390351 | 1 | 0 |
| 090 | - | 31111 | 0 | 31111 | 3390351 | 3421462 | 0 | 0 |
| 126 | - | 231 | 0 | 231 | 3421462 | 3421693 | 0 | 0 |
| 119 | - | 121 | 0 | 121 | 3421693 | 3421814 | 1 | 0 |
| 073 | + | 3010 | 0 | 3010 | 3421815 | 3424825 | 4 | 10 |
| 113 | + | 85 | 0 | 85 | 3424833 | 3424918 | 0 | 0 |
| 104 | - | 864 | 9 | 864 | 3424919 | 3425774 | 7 | 0 |
| 039 | - | 818 | 0 | 818 | 3425783 | 3426601 | 4 | 0 |
| 103 | + | 210 | 0 | 210 | 3426601 | 3426811 | 5 | 0 |
| 043 | + | 119 | 0 | 119 | 3426811 | 3426930 | 0 | 0 |
| 121 | - | 78 | 0 | 78 | 3426931 | 3427011 | 1 | 2 |
| 004 | + | 41173 | 0 | 41173 | 3427011 | 3468184 | 0 | 0 |
| 005 | + | 1031 | 0 | 1031 | 3468185 | 3469216 | 2 | 0 |
| 086 | + | 112233 | 0 | 112233 | 3469216 | 3581450 | 0 | 1 |
| 117 | - | 768 | 0 | 768 | 3581450 | 3582218 | 0 | 0 |
| 054 | - | 35079 | 0 | 35079 | 3582218 | 3617298 | 0 | 1 |
| 101 | + | 136 | 0 | 136 | 3617298 | 3617434 | 0 | 0 |
| 084 | - | 733 | 0 | 733 | 3617434 | 3618167 | 0 | 0 |
| 087 | - | 1470 | 0 | 1470 | 3618167 | 3619637 | 1 | 0 |
| 025 | - | 598 | 0 | 598 | 3619637 | 3620235 | 0 | 0 |
| 122 | - | 86 | 0 | 86 | 3620235 | 3620321 | 1 | 0 |
| 123 | - | 65 | 0 | 65 | 3620321 | 3620386 | 2 | 0 |
| 118 | - | 29673 | 0 | 29673 | 3620386 | 3650058 | 0 | 1 |
| 116 | - | 1195 | 0 | 1195 | 3650058 | 3651253 | 0 | 0 |
| 032 | - | 12974 | 0 | 12974 | 3651253 | 3664227 | 0 | 0 |
| 047 | + | 1133 | 0 | 1133 | 3664227 | 3665360 | 0 | 0 |
| 046 | + | 94930 | 0 | 94930 | 3665360 | 3760289 | 0 | 1 |
| 101 | + | 136 | 0 | 136 | 3760289 | 3760425 | 0 | 0 |
| 084 | - | 733 | 0 | 733 | 3760425 | 3761158 | 0 | 0 |
| 087 | - | 1470 | 0 | 1470 | 3761158 | 3762628 | 0 | 0 |
| 025 | - | 598 | 0 | 598 | 3762628 | 3763226 | 0 | 0 |
| 122 | - | 86 | 0 | 86 | 3763226 | 3763312 | 1 | 0 |
| 120 | + | 176160 | 0 | 176160 | 3763312 | 3939470 | 0 | 4 |
| 115 | - | 214 | 0 | 214 | 3939470 | 3939684 | 2 | 0 |
| 043 | - | 119 | 0 | 119 | 3939684 | 3939803 | 0 | 0 |
| 103 | - | 210 | 0 | 210 | 3939803 | 3940013 | 5 | 0 |
| 039 | + | 818 | 0 | 818 | 3940013 | 3940831 | 1 | 0 |
| 040 | + | 561 | 0 | 561 | 3940831 | 3941392 | 0 | 0 |
| 114 | - | 298 | 0 | 297 | 3941393 | 3941604 | 4 | 126 |
| 113 | - | 85 | 0 | 85 | 3941604 | 3941689 | 0 | 0 |
| 100 | - | 16 | 0 | 16 | 3941689 | 3941705 | 0 | 0 |
| 073 | - | 3010 | 0 | 3010 | 3941706 | 3944871 | 8 | 155 |
| 105 | - | 88477 | 0 | 88477 | 3944716 | 4033193 | 1 | 0 |
| 115 | - | 214 | 0 | 214 | 4033193 | 4033407 | 0 | 0 |
| 043 | - | 119 | 0 | 119 | 4033407 | 4033526 | 0 | 0 |
| 103 | - | 210 | 0 | 210 | 4033526 | 4033736 | 1 | 0 |
| 039 | + | 818 | 0 | 818 | 4033736 | 4034554 | 1 | 0 |
| 104 | + | 864 | 9 | 864 | 4034563 | 4035418 | 9 | 0 |
| 113 | - | 85 | 0 | 85 | 4035419 | 4035504 | 0 | 0 |
| 100 | - | 16 | 0 | 16 | 4035504 | 4035521 | 0 | 1 |
| 073 | - | 3010 | 0 | 3010 | 4035521 | 4038533 | 5 | 4 |
| 119 | + | 121 | 0 | 121 | 4038534 | 4038655 | 1 | 0 |
| 058 | - | 213 | 0 | 213 | 4038655 | 4038868 | 0 | 0 |
| 045 | + | 125147 | 0 | 125147 | 4038868 | 4164320 | 0 | 311 |
| 115 | - | 214 | 0 | 214 | 4164321 | 4164535 | 0 | 0 |
| 043 | - | 119 | 0 | 119 | 4164535 | 4164654 | 0 | 0 |
| 103 | - | 210 | 0 | 210 | 4164654 | 4164864 | 1 | 0 |
| 039 | + | 818 | 0 | 818 | 4164864 | 4165682 | 0 | 0 |
| 040 | + | 561 | 0 | 561 | 4165682 | 4166243 | 1 | 0 |
| 114 | - | 298 | 0 | 298 | 4166243 | 4166541 | 0 | 0 |
| 113 | - | 85 | 0 | 85 | 4166541 | 4166626 | 1 | 0 |
| 100 | - | 16 | 0 | 16 | 4166626 | 4166643 | 0 | 1 |
| 073 | - | 3010 | 0 | 3010 | 4166643 | 4169653 | 8 | 10 |
| 119 | + | 121 | 0 | 121 | 4169654 | 4169775 | 0 | 0 |
| 058 | - | 213 | 0 | 213 | 4169775 | 4169988 | 1 | 0 |
| 003 | + | 4113 | 0 | 4113 | 4169988 | 4174101 | 0 | 0 |
| 005 | - | 1031 | 0 | 1031 | 4174101 | 4175132 | 0 | 0 |
| 024 | + | 30812 | 0 | 30812 | 4175132 | 4205944 | 0 | 0 |
| 121 | + | 78 | 0 | 78 | 4205944 | 4206022 | 0 | 0 |
| 043 | - | 119 | 0 | 119 | 4206023 | 4206142 | 0 | 0 |
| 103 | - | 210 | 0 | 210 | 4206142 | 4206352 | 0 | 0 |
| 039 | + | 818 | 0 | 818 | 4206352 | 4207170 | 0 | 0 |
| 040 | + | 561 | 0 | 561 | 4207170 | 4207731 | 0 | 0 |
| 114 | - | 298 | 0 | 297 | 4207732 | 4207943 | 3 | 126 |
| 113 | - | 85 | 0 | 85 | 4207943 | 4208028 | 0 | 0 |
| 100 | - | 16 | 0 | 16 | 4208028 | 4208045 | 0 | 1 |
| 073 | - | 3010 | 0 | 3010 | 4208045 | 4211056 | 9 | 1 |
| 119 | + | 121 | 0 | 121 | 4211057 | 4211178 | 0 | 0 |
| 057 | - | 284276 | 0 | 284276 | 4211178 | 4496203 | 2 | 751 |
| 053 | - | 625 | 0 | 625 | 4496203 | 4496828 | 0 | 0 |
| 011 | + | 706 | 0 | 706 | 4496828 | 4497534 | 0 | 0 |
| 075 | + | 7946 | 0 | 7946 | 4497534 | 4505481 | 0 | 1 |
| 111 | - | 181 | 0 | 181 | 4505481 | 4505662 | 0 | 0 |
| 080 | - | 1041 | 0 | 1041 | 4505662 | 4506702 | 0 | 1 |
| 079 | - | 148241 | 15389 | 148241 | 4506702 | 4639675 | 0 | 123 |
