# Supplementary material for: OMACC: an Optical-Map-Assisted Contig Connector for improving de novo genome assembly
Source: BMC Syst Biol. 2013 Dec 13;7(Suppl 6):S7. doi: 10.1186/1752-0509-7-S6-S7 (PMC4029551; doi:10.1186/1752-0509-7-S6-S7)
Supplement: Additional file 2 — Table S2. Order of E. coli contigs inferred from the SOMA2 alignments and the mapping status (U: unique, N: non-unique). [file 1752-0509-7-S6-S7-S2.docx]

| Contig | Mapping |
| --- | --- |
| 063+ | N |
| 031+ | U |
| 020- | U |
| 055- | U |
| 038- | U |
| 081+ | N |
| 052- | N |
| 076+ | U |
| 029- | U |
| 010+ | U |
| 069- | U |
| 090+ | N |
| 026+ | N |
| 108+ | U |
| 062- | U |
| 016+ | U |
| 033- | U |
| 060+ | N |
| 085+ | U |
| 112+ | N |
| 066+ | N |
| 048+ | U |
| 044- | U |
| 017+ | U |
| 012- | U |
| 036- | U |
| 001+ | U |
| 023+ | U |
| 022+ | U |
| 002+ | U |
| 050- | U |
| 059- | U |
| 107- | U |
| 013+ | U |
| 061+ | U |
| 006+ | U |
| 091+ | U |
| 067- | U |
| 019- | U |
| 004+ | U |
| 086+ | U |
| 054- | U |
| 118- | N |
| 032- | U |
| 046+ | U |
| 120+ | U |
| 105- | U |
| 045+ | U |
| 057- | U |
| 079- | U |
