# Supplementary material for: OMACC: an Optical-Map-Assisted Contig Connector for improving de novo genome assembly
Source: BMC Syst Biol. 2013 Dec 13;7(Suppl 6):S7. doi: 10.1186/1752-0509-7-S6-S7 (PMC4029551; doi:10.1186/1752-0509-7-S6-S7)
Supplement: Additional file 3 — Table S3. Order of GI1 contigs inferred from the SOMA2 alignments and the mapping status (U: unique, N: non-unique). [file 1752-0509-7-S6-S7-S3.docx]

| Contig | Mapping |
| --- | --- |
| 041+ | U |
| 104+ | U |
| 074+ | N |
| 030- | U |
| 132+ | U |
| 014+ | U |
| 069+ | N |
| 076- | U |
| 046- | U |
| 119+ | U |
| 018- | U |
| 033- | U |
| 082+ | U |
| 037+ | U |
| 025+ | U |
| 066- | U |
| 086- | U |
| 022- | U |
| 026+ | U |
| 044+ | U |
| 020- | U |
| 068+ | U |
| 048+ | N |
| 060- | U |
| 036+ | U |
| 013- | U |
| 001- | U |
| 017+ | U |
| 043- | U |
| 056+ | U |
| 005+ | U |
| 008- | U |
| 063+ | N |
| 087- | U |
| 084+ | U |
| 071+ | U |
| 027- | U |
| 090+ | N |
| 106+ | N |
| 016- | U |
| 052+ | U |
| 015+ | U |
| 021- | U |
| 051+ | U |
| 003+ | U |
| 038+ | U |
| 019- | U |
| 034+ | U |
| 007+ | U |
| 039- | U |
| 057- | U |
| 042+ | U |
| 091+ | N |
| 049- | U |
| 078+ | U |
| 029- | U |
| 070- | U |
| 055- | U |
| 040+ | U |
| 011+ | U |
| 050- | U |
| 075- | U |
| 023+ | U |
| 010+ | U |
| 002+ | U |
| 004- | U |
| 035+ | U |
| 111+ | N |
| 006- | U |
